# Adapting Yoga Therapy to Meet the Needs of Inpatients Undergoing Hematopoietic Stem Cell Transplantation: Insights From an International, Multisite, Qualitative Study

**DOI:** 10.1200/GO-25-00567

**Published:** 2026-03-06

**Authors:** Smitha Mallaiah, Vijaya Majumdar, Scherezade K. Mama, Amanda L. Olson, May Daher, Yamuna Badgare, Nataraj Kolar Srinivasrao, Stella Rwezaula, Peter Muhoka, Raghavendra Rao, Sachin Jadav, Richard Wagner, Anna Wilson, Manjunath Nandi Krishnamurthy, Lorenzo Cohen

**Affiliations:** ^1^ The University of Texas MD Anderson Cancer Center, Houston, TX; ^2^ Swami Vivekananda Yoga Anusandhana Samsthana (S-VYASA), Bengaluru, India; ^3^ Health Care Global—HCG, Bengaluru, India; ^4^ Muhimbili National Hospital, Dar es Salaam, Tanzania

## Abstract

**PURPOSE:**

Yoga therapy (YT) is a growing mind-body approach in oncology. However, its integration into hematopoietic stem cell transplantation (HSCT) remains limited, particularly in non-Western and resource-constrained settings. This formative qualitative study aimed to explore the lived experiences of HSCT patients in the United States, India, and Tanzania to inform the development of a culturally adapted YT protocol.

**METHODS:**

Fifteen post-HSCT adult participants were recruited from three international cancer centers using purposive sampling. Semistructured interviews were conducted in participants' preferred languages, transcribed, and thematically analyzed by an interdisciplinary team. Themes were mapped to domains of the Adaptome framework to guide adaptation decisions.

**RESULTS:**

Across sites, fatigue, sleep disruption, isolation, and emotional distress were common. Participants expressed interest in supportive, noninvasive interventions like yoga, but familiarity varied by region. Cultural adaptations included neutral, nonreligious language for meditation, flexible session timing, caregiver inclusion, same-language preference, and same-sex therapist. Contextual adaptations addressed environmental limitations (eg, space constraints, infection precautions), such as bed- and chair-based modules. Participants recommended short (20-30-minute), afternoon sessions with hybrid delivery options. Trust in the person introducing yoga (eg, physician *v* therapist) emerged as a culturally relevant factor.

**CONCLUSION:**

This formative study offers critical insights into adapting (YT) for patients undergoing HSCT across diverse global contexts. Using the Adaptome framework, we applied a systematic, culturally, and contextually responsive approach that preserved core therapeutic elements while enhancing feasibility and relevance. These evidence-based adaptations directly informed the design and implementation of a single-arm, phase II trial of adjunct yoga in HSCT patients.

## INTRODUCTION

As there is recognition globally of the importance of supportive interventions that address patients' psychological, emotional, and physical well-being, culturally adapted interventions are a promising strategy for improving health outcomes, particularly in diverse and underserved populations. Patients undergoing hematopoietic stem cell transplantation (HSCT) experience a challenging treatment process associated with significant short- and long-term physical and psychological burdens.^1^ HSCT is often regarded as the most challenging form of cancer treatment and often leads to posttraumatic stress disorder symptoms.^2^ Yet, culturally tailored interventions in this setting remain scarce and program effectiveness does not always translate directly across different cultural or health care environments.^3,4^ Intentional adaptation, whether proactive or in response to emerging barriers, is increasingly recognized as a more efficient and scalable approach.^5^

**CONTEXT**

**Key Objective**
Can yoga therapy (YT) be adapted to address the physical and emotional challenges of hematopoietic stem cell transplantation (HSCT) patients across diverse, resource-constrained global settings?
**Knowledge Generated**
In a qualitative study of 15 post-HSCT participants in the United States, India, and Tanzania, fatigue, sleep disruption, isolation, and emotional distress were common. Using the Adaptome framework, a systematic, culturally and contextually responsive YT protocol was developed. Participants endorsed yoga as a supportive, noninvasive intervention but emphasized region-specific needs: culturally neutral language, flexible timing, same-sex therapists, and adaptations for space and infection constraints.
**Relevance**
Integrative medicine approaches like YT can improve supportive care for HSCT patients, including in low- and middle-income countries, by offering accessible, low-cost strategies to reduce treatment-related symptom burden and enhance quality of life globally.

Evidence also suggests that individualized, need-based, and culturally tailored interventions are more effective in improving engagement and outcomes.^6^ One such promising modality to improve multiple aspects of quality of life in oncology is yoga. Although it originated in India, yoga is widely practiced. Yoga is recommended on (ASCO‑Society for Integrative Oncology) and National Comprehensive Cancer Network clinical care guidelines for patients with cancer to help manage mood, fatigue, pain, and other symptoms commonly experienced by patients with cancer and those undergoing HSCT.^7-9^ A culturally adapted yoga protocol for HSCT patients is timely, given yoga's origins and therapeutic value.^10-12^

The goal of cultural adaptation is to enhance the relevance, acceptability, and effectiveness of interventions by integrating cultural patterns, meanings, and values.^13^ Research shows that culturally adapted interventions are significantly more effective for ethnically underrepresented patients than nonadapted ones.^14,15^ However, few supportive care interventions have been culturally adapted, piloted, or evaluated within the context of cancer care.^16^ This is especially true of mind-body interventions in the field of integrative medicine.

Cultural adaptation of a yoga intervention involves modifying an evidence-based protocol to incorporate culturally sensitive elements such as language, social norms, and local values that align with patients' worldviews. These adaptations may range from surface-level changes (eg, translating materials into preferred languages) to deep-level modifications (eg, integrating sociocultural values such as family involvement in collectivist cultures or using culturally resonant metaphors and proverbs). Systematic reviews of culturally adapted psychological interventions show that cultural adaptations often lead to improved outcomes and higher retention rates, particularly among racially and ethnically underserved populations.^17^ However, significant gaps remain in the adaptation and implementation of integrative interventions, including yoga in cancer care, especially among patients undergoing HSCT.

The primary objective of this qualitative study was to explore the lived experiences of patients who underwent an HSCT and gather their feedback on the integration of yoga during treatment while hospitalized and after discharge. Specifically, we aimed to understand perceived risks and barriers, as well as identifying social, environmental, and cultural factors that influence both initial engagement and long-term adherence. The secondary objective was to identify key characteristics necessary for designing and testing a culturally sensitive yoga therapy (YT) program tailored to adult patients with cancer across three distinct health care systems and cultures: the United States, India, and Tanzania.

## METHODS

### Study Design and Participants

We conducted a qualitative study using semistructured interviews to explore patient experiences, symptom burden, cultural preferences, and practical considerations for designing a YT program for individuals undergoing HSCT. The adaptation process was guided by the Adaptome framework,^18^ to align the intervention with diverse cultural and clinical contexts. Participants were recruited from three international cancer centers: The University of Texas MD Anderson Cancer Center (MDA), Houston, TX; HealthCare Global Enterprises (HCG), Bengaluru, India; and Muhimbili National Hospital (MNH), Dar es Salaam, Tanzania.

### Study Settings

#### MDA, USA

MDA in Houston, Texas, is a National Cancer Institute-designated comprehensive cancer center, performing 750 transplants annually. Multidisciplinary integrative services, such as massage, music therapy, nutrition, psycho-oncology, and physiotherapy, are available.

#### HCG, India

HCG is South Asia's largest oncology network, with over 18 centers in India, including one center in Africa, treating 100,000 new patients annually. The Bangalore center performs about 80 transplants per year. Supportive care services, including nutrition, psycho-oncology, and physiotherapy, are offered. The hospital is nationally accredited and equipped with advanced oncology facilities.

#### MNH, Tanzania

MNH is Tanzania's largest public referral and teaching hospital, in Dar es Salaam, Tanzania. It provides comprehensive care, including oncology, to approximately 4,000 patients daily. In partnership with HCG, MNH performs up to 12 autologous stem cell transplants annually. Patients stay in HSCT units, and access to supportive or integrative services, such as yoga or psychosocial care, is unavailable.

### Participant Recruitment and Sampling

We used purposive sampling to ensure diversity in sex, age, transplant type (autologous *v* allogeneic), and sociocultural background. At each site, hematology-oncology physicians identified patients who had undergone HSCT recently. All interviews were conducted between day 100 and day 437 post-HSCT. Study coordinators contacted eligible patients, introduced the study, and obtained both written and verbal informed consent from those who agreed to participate.

### Ethical Approval

This study was approved by the Institutional Review Board at MDA Cancer Center (Protocol No. 2022-0785) and by the ethics committees of HCG and MNH.

### Interview Procedures

Participants completed one 35-60-minute semistructured interview via Zoom. Interviews in the United States and India were conducted in English by a YT specialist with clinical experience in cancer care. One interview in India was conducted in Hindi. In Tanzania, interviews were conducted by the treating physician, with the yoga therapist present, and interviews were conducted in English or Swahili. All non-English responses were translated into English by bilingual staff and verified by a second team member. No additional procedures or interventions were performed beyond the interview itself.

### Adaptome Framework and Interview Guide

**FIG 1:**
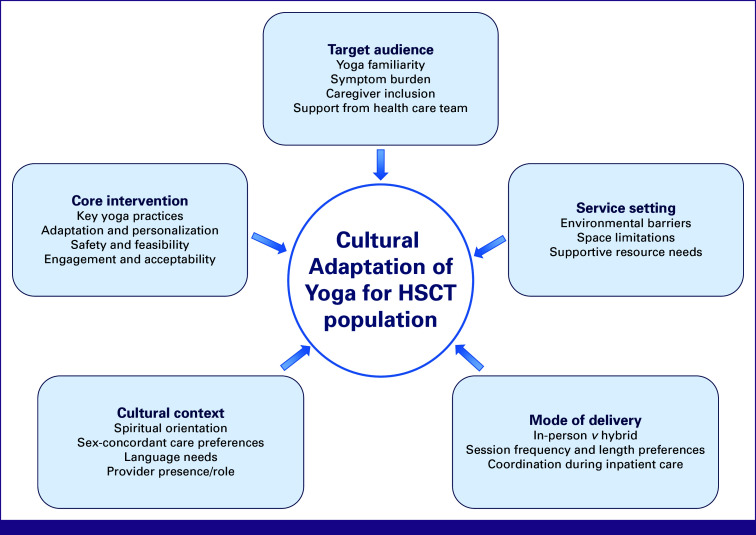
Adaptome framework domains for the cultural adaptation of yoga therapy. HSCT, hematopoietic stem cell transplantation.

Development of the interview guide was guided by the Adaptome framework,^18^ which identifies sources of adaptation and how interventions may be systematically modified across populations, settings, and contexts while maintaining effectiveness (Fig 1). Key adaptation domains explored included target audience (eg, age, health literacy), service settings (physical barriers, space, resources), mode of delivery (eg, session length, mode), cultural context (eg, values, norms, worldview), and core intervention components (intervention elements essential for efficacy, individualization).

The guide was developed with input from a collaborator experienced in cultural adaptation. The lead interviewer had experience with HSCT inpatients and expertise in YT across diverse populations. Given that interviewers were yoga therapists, reflexivity and structured neutrality were emphasized throughout data collection to minimize potential bias toward positive perceptions and data analysis was reviewed by a researcher without a yoga background to enhance objectivity.

The semistructured guide covered the following topics:

- Patients' experiences with HSCT, including its associated symptom burden and physical and psychological coping strategies (eg, rest, prayer, movement),
- Familiarity with and attitudes toward yoga and meditation,
- Perceived acceptability of proposed yoga practices (gentle movement, breathing, meditation),
- Preferences regarding session frequency, duration, and delivery mode (in-person, online, hybrid),
- Cultural considerations, including language, therapist characteristics, representation in materials, and accessibility.

### Data Collection and Analysis

Demographic data were collected and analyzed descriptively. All interviews were audio/video recorded, transcribed verbatim, deidentified, and securely stored. Thematic analysis was conducted using an inductive approach. Two researchers independently coded the transcripts to identify initial codes and develop a preliminary coding framework, which was refined iteratively through team discussions. A third team member reviewed and reconciled discrepancies through iterative discussions until consensus on code definitions and thematic structure was reached. Recruitment continued until thematic saturation was reached, defined as the point at which no new themes emerged within each site and subsequently across all three centers. Credibility and rigor were ensured through triangulation, peer debriefing, and maintenance of a detailed audit trail.

**FIG 2:**
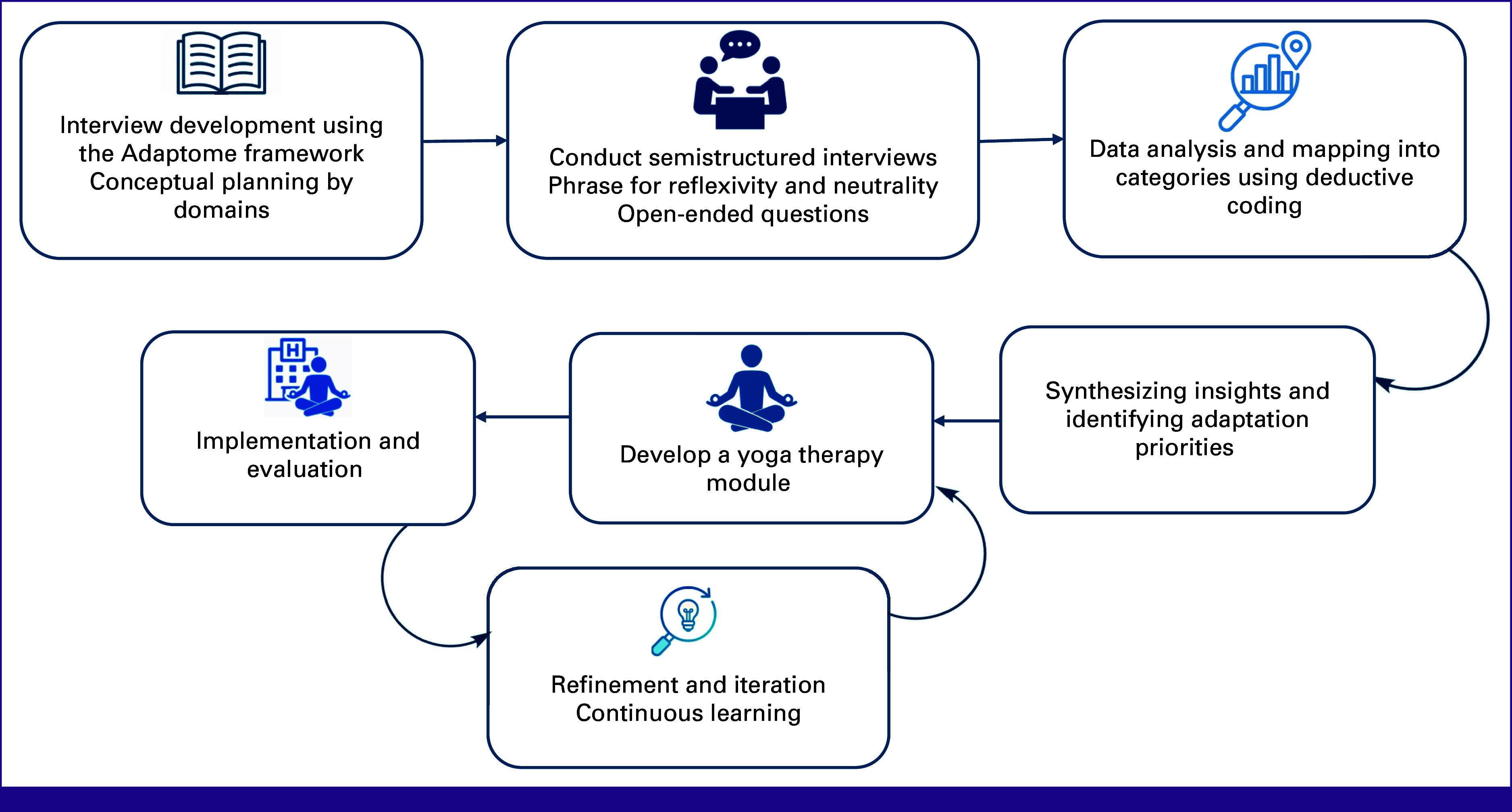
Schematic representation of the Adaptome-guided yoga therapy adaptation and implementation process.

Following thematic analysis, emergent themes and subthemes were systematically mapped to the five Adaptome domains to guide contextual adaptation. Mapping was based on the functional locus of change, who the adaptation affects, where/by whom it is delivered, how it is delivered, why/with what cultural meaning, or what active ingredients are modified. Two investigators independently assigned each theme to its primary domain and resolved discrepancies by consensus (eg, physical space constraints mapped to service setting; language preferences mapped to cultural context). Figure 2 displays the schematic representation of the Adaptome-guided process used to adapt the YT intervention

## RESULTS

### Participant Characteristics

Fifteen participants were interviewed, five at each of the three sites. At both MDA and HCG, five of six eligible patients consented and enrolled (83.3%), while all five patients approached at MNH consented and enrolled (100%). Participant age ranged from 29 to 71 years. Each site included three men and two women. MDA participants were racially and ethnically diverse (Hispanic/Latino, White, Black), whereas cohorts at HCG and MNH were racially and ethnically homogeneous, comprising Indian and Black African patients, respectively. Diagnoses included multiple myeloma and Hodgkin lymphoma, with both autologous and allogeneic transplant (at MNH, all were autologous). The mean number of days since transplant across three centers was 234.6 (range ±135.17). Educational backgrounds varied, and most were married. See Table 1 for full demographic information.

**TABLE 1:** Demographic and Clinical Characteristics of Participants by Study Site

Characteristic	MDA (n = 5), No. (%)	HCG (n = 5), No. (%)	MNH (n = 5), No. (%)
Enrollment rate	5/6 (83.3)	5/6 (83.3)	5/5 (100)
Age, mean (range)	53.2 (29-71)	39.2 (33-52)	46.0 (34-57)
Sex			
Male	3 (60)	3 (60)	3 (60)
Female	2 (40)	2 (40)	2 (40)
Ethnicity			
Hispanic/Latino	2 (40)	0	0
White/Caucasian	2 (40)	0	0
Black	1 (20)	0	0
Indian	0	5 (100)	0
African	0	0	5 (100)
Diagnosis			
Multiple myeloma	3 (60)	0	4 (80)
Hodgkin lymphoma	0	3 (60)	1 (20)
AML	1 (20)	0	0
Myelodysplastic syndrome	1 (20)	0	0
ALL	0	1 (20)	0
Myelomonocytic leukemia	0	1 (20)	0
Transplant type			
Autologous	3 (60)	3 (60)	5 (100)
Allogeneic	2 (40)	2 (40)	0
Days since transplant, mean (SD)	149.4 (±126.22)	250.4 (±121.80)	304 (±133.34)
Marital status			
Married	3 (60)	4 (80)	4 (80)
Unmarried/single	2 (40)	1 (20)	1 (20)
Educational attainment			
Primary education	0	0	1
Secondary/high school	0	1	2
Associate degree	1	0	0
Bachelor's degree	4	1	2
Master's degree	0	3	0

Abbreviations: HCG, HealthCare Global; MDA, MD Anderson Cancer Center; MNH, Muhimbili National Hospital; SD, standard deviation.

Across three centers, patients reported significant physical and psychological symptom burden following HSCT. They employed diverse coping strategies, and many patients also reflected on past experiences of stress, drawing on those experiences to navigate their current challenges.

### Symptom Burden and Experiences During HSCT

Across all sites, fatigue emerged as the most frequently reported and burdensome symptom, affecting participants' physical functioning and sense of normalcy. Participants described this fatigue as crippling and completely draining, affecting their ability to walk, eat, or engage in daily routines. Other common symptoms included nausea, vomiting, mucositis, and gastrointestinal disturbances. Several participants also described sleep disruption and psychological distress, including anxiety, isolation, and fear of complications. “When I had an infection, I was confined to my room; it felt like jail” (MDA04).

Although many symptoms were common across settings, site-specific challenges emerged. At MDA, one patient experienced hemorrhagic cystitis that required catheterization, which was described as mentally exhausting (MDA02). A patient at HCG (HCG01) reported an ICU admission that created significant fear and uncertainty. At MNH, participants noted limited pretransplant preparation and psychosocial support: “We didn't really know what to expect” (MNH05).

Participants' narratives highlighted psychological impact of visible physical changes. One participant at MDA reflected, “To actually have to lose all your hair with one chemo was just traumatic” (MDA03). At HCG, a patient described the emotional toll of incontinence due to treatment side effects: “I had to be in a diaper… Mentally, you know, your body is not accepting the diaper” (HCG02). At MNH, sleep disturbance was distressing, as one participant recalled pleading with physicians for medication to sleep: “It was very difficult… I even begged the doctors” (MNH01).

### Coping Strategies Across Sites

Three overarching coping strategies emerged from participant interviews: spiritual coping, psychosocial support, and engagement in stress-reducing activities.

Spiritual coping, including prayer, was the dominant coping strategy, particularly at MNH, where it formed the foundation of patients' emotional resilience. Participants described daily prayer to find meaning, hope, and strength. At HCG and MDA, spiritual coping surfaced frequently, often intertwined with gratitude, acceptance, and belief in divine healing. One HCG participant explained, “I had to trust in God… That’s the only thing that gave me peace” (HCG02).

Psychosocial support from spouses, family, and close friends was critical to emotional well-being. At MDA and HCG, patients frequently cited partners or adult children as key sources of strength. Frequent video calls with family, especially grandchildren, were described as “kept me going” and “the only thing that brought happiness.” At MNH, although less explicitly discussed, family and community support were present, often embedded within spiritual practices or community-based coping.

Engagement in stress management activities, such as walking, reading, listening to music, or chess, also supported psychological well-being, although this theme was less prevalent at MNH. Participants at MDA and HCG described these activities as helping them feel normal again or offering a mental escape from the clinical environment. Others described reframing thoughts through gratitude or positive outcomes, which helped maintain emotional stability during hospitalization. Table 2 provides a summary of coping strategies and representative quotes.

**TABLE 2:** Coping Themes and Subthemes Across Sites

Main Theme	Subthemes	MDA	HCG	MNH
Spiritual coping	Daily prayer rituals Faith in spiritual or/religious sources Seeking meaning and purpose Hope for healing Acceptance in difficult situations	Frequently relied on prayer and personal faith. Quotes: “I'm a Christian and so prayer is meditation for me” (MDA01) “Faith and prayer can provide emotional relief” (MDA05)	Prayer and spiritual practices were central to emotional coping Quotes: “…prayer is the first thing” (HCG04) “When things got really hard, I just kept praying” (HCG02)	Consistently reported strategy, primary means of coping Quotes: “I entrusted all matters to God” (MNH02) “…think prayer helped me a lot” (MNH05)
Social support	Family support Peer support Professional support Virtual connection (eg, video calls)	Strong support from spouse, family, and friends Quote: “Support from the right people can make even the hardest battles more bearable” (MDA02)	Family and friends are described as critical Quotes: “My spouse was a great support…” (HCG04) “My sister was there, and she was cheering me up” (HCG03)	Less explicitly emphasized, present as a background factor Quote**:** “I talked with family…” (MNH01)
Engagement in activities	Physical engagement (exercise, walking, gardening) Recreational engagement (TV, music, reading, chess) Cognitive engagement (positive thinking, mental stimulation)	Wide range of physical and recreational activities Quotes: “Every small step forward is a step toward healing” (MDA03) “It's helpful, especially for my morale, knowing that I've accomplished something” (MDA04)	Recreational and physical activities are used to maintain normalcy Quotes: “Playing chess gives me the same kind of experience to focus…” (HCG03) “Healing is not just about medicine; it's about mindset” (HCG01)	Meaningful engagement with music, walking, and positive thinking Quotes: “I like imagining myself in the future, in a better situation (MNH 04) “I prefer movement and exercise …” (MNH03)

Abbreviations: HCG, HealthCare Global; MDA, MD Anderson Cancer Center; MNH, Muhimbili National Hospital.

### Cultural and Contextual Adaptation of YT

Guided by the Adaptome framework, emergent qualitative themes were inductively derived from participant narratives and then deductively aligned with Adaptome domains to identify modifiable areas for program tailoring based on culture, context, and systemic factors. Participants across all three sites expressed openness to a structured program focused on gentle movement, breathing, and meditation, but emphasized that successful implementation would require adaptation to the realities of their clinical and cultural contexts. Table 3 integrates the data-to-framework mapping, illustrating how themes were linked to specific Adaptome domains and corresponding adaptation recommendations.

**TABLE 3:** Data-to-Framework Mapping and Cultural Adaptations Across Adaptome Domains

Adaptome Area	Themes and Subthemes	Related Cultural/Contextual Values	Adaptations or Recommendations
Target audience	Familiarity with yoga and meditation (HCG: high; MDA: moderate; MNH: low)	Cultural exposure varied by site	Provide orientation across all sites
	Desire for movement, normalcy, effective coping strategies	Movement fosters control, identity, and coping	Emphasize movement-based practices and their psychosocial benefits
	Symptom burden and physical variability	Autonomy and self-awareness of limits	Provide flexible, modular programming with optional participation
	Include caregiver/spouse	Connection to family	Invite caregivers with patient approval
Service setting	Physical barriers: Limited space, IV lines, hygiene concerns	Space constraints and resource limitations in LMIC	Implement infection-safe practices
	Requests for supportive resources (massage, reading, counseling)	Holistic well-being in limited-resource settings	Incorporate massage therapy, resource libraries, counseling, and supportive services where feasible
	“Who Should Introduce Yoga” (doctors preferred overall; HCG open to yoga therapists)	Trust in the medical hierarchy	Tailor introduction per site: involve doctors initially, with yoga therapists providing follow-up
Mode of delivery	Delivery preference: (53% in-person, 47% hybrid)	Connection, support, flexibility	Default to in-person, with hybrid options for continuity
	Preferred session timing: 20-30 minutes, afternoon, three times per week to daily	Adaptive coping	Align sessions with patient-preferred timing and frequency
	Interruptions during meditation	Hospital routines disrupt practice	Coordinate with nursing staff to reduce interruptions
	Introduce yoga 2-3 weeks pretransplant	Preparation, empowerment, familiarity	Begin yoga education and practice before transplant
	Long-term engagement important to sustain yoga practice	Yoga as a recovery tool	Develop postdischarge yoga pathways and referral systems
Cultural context	For some, meditation was equated with prayer	Spiritual practices may substitute for structured mindfulness	Use accessible language that honors spiritual and cultural meanings
	Preference for same-sex yoga therapist	Modesty, privacy, and/or sex concordance	Offer clients the option to choose a same-sex therapist; document preferences during intake
	Importance of therapist presence	Emotional support during inpatient care	Prioritize in-person sessions with therapist support
	Language preference: MDA, English; HCG, flexible; MNH, Swahili	Effective communication and cultural respect	Use local languages or bilingual instructors as needed
Core intervention components	Openness to engaging in yoga therapy components, including gentle movement, pranayama, and meditation	Patient-centered care	Educate on yoga benefits and provide materials (video, handouts) for self-practice
	Symptom burden, fatigue, safety risk	Need for safety, energy conservation, and respect for physical limitations	Adapted movement, breathwork, and meditation via chair- and/or bed-based modules with pose modifications, safety, and self-awareness

NOTE. Themes were inductively derived from qualitative interviews and mapped onto Adaptome domains to guide culturally and contextually relevant adaptations.

Abbreviations: HCG, HealthCare Global; LMIC, low- and middle-income countries; MDA, MD Anderson Cancer Center; MNH, Muhimbili National Hospital.

Under the target audience, familiarity with yoga varied widely. HCG patients described previous exposure but noted they had not practiced recently. MDA participants had a general understanding of yoga's components but varied in confidence. MNH participants were largely unfamiliar with yoga. Across all sites, participants emphasized the importance of a clear introduction to yoga, what it is, and what it entails before initiating practice. Many participants noted that movement could help break the monotony, reduce fatigue, and offer a sense of control during an otherwise restrictive period. Inclusion of family members was especially valued at HCG and MNH, “yoga should be offered to spouses/caregivers, my wife was overwhelmed caring for me” (HCG01).

Service setting constraints included limited space, particularly at HCG and MNH; “You're stuck in a small room all day, you just want to get out” (HCG04). HSCT itself also adds to concerns of hygiene and infection control, with concerns of using the floor for yoga and medical equipment such as IV lines across all three centers. These realities shaped patient preferences for modified bed- or chair-based movement. Patients at HCG and MNH also expressed interest in additional supportive services such as massage, psychosocial counseling, and reading materials, to address broader gaps in supportive resources.

Regarding mode of delivery, most participants preferred in-person sessions, though hybrid or video-based options were seen as acceptable. Afternoon sessions lasting 20-30 minutes were preferred, with suggested frequency ranging from three times per week to daily, depending on symptom severity and patient capability. Participants emphasized the need to coordinate with staff to avoid interruptions during sessions.

Cultural context and linguistic adaptation were crucial, especially in settings where yoga might be conflated with religious practices. Some participants emphasized the need to frame meditation and breathing practices using secular and inclusive language. Patients at MNH and MDA preferred a medical professional (e.g., doctor) to introduce the intervention. A patient said, “I'd feel more confident joining if my doctor introduced the yoga study first,” (MNH03), while HCG participants were open to a medical professional or trained yoga therapists. Language preferences highlighted the importance of using local or bilingual instruction to ensure understanding. At MNH, all patients preferred a yoga therapist of the same sex, “I’d be more comfortable if the yoga therapist was male, it’s easier to mentally open up…” said a male patient (MNH01). At HCG, both the female patients preferred a female therapist “I prefer a female yoga therapist, it’s just more comfortable” (HCG05). Patients at MDA have expressed that they do not have a specific preference regarding the sex of their provider. Across all sites, patients emphasized the importance of having a yoga therapist who is knowledgeable, kind, and empathetic toward their symptom burden.

Core intervention components of gentle movement, breathwork, meditation, and relaxation were widely accepted, provided they were adapted for patient energy levels and treatment stage. “Some days, I was so exhausted I couldn't even think about getting out of bed” (HCG03). Patients valued clear explanations of the benefits and reassurance about safety, especially when immunocompromised or physically limited. Flexibility and personalization were viewed as essential for maintaining participation and ensuring long-term acceptability.

## DISCUSSION

To our knowledge, this is the first study to explore cultural and contextual adaptations of yoga-based interventions for HSCT patients across three diverse global settings: India, Tanzania, and the United States Moreover, no previous studies have systematically conducted research to adapt yoga for HSCT patients, let alone ensuring culturally sensitive adaptations. By systematically exploring the Adaptome components, our findings provide a structured blueprint for integrating complementary therapies into complex oncology care across diverse settings.

Among the key themes that emerged were patients' coping mechanisms, which offer valuable insights into how yoga-based interventions can be meaningfully tailored. Notably, spirituality was identified as the primary coping mechanism among patients across all three centers, underscoring the critical need to incorporate spiritual components into care to address the culture-specific needs of diverse patient populations.^19^

Our findings reinforce the importance of culturally and contextually tailored supportive care in oncology, particularly in the HSCT setting, where intensive treatment protocols and infrastructure constraints often limit patient engagement in nonpharmacological therapies. Service-level adaptations, such as infection control practices, reflect well-documented feasibility challenges in HSCT environments, where immunosuppression and fatigue restrict participation in traditional yoga formats.^20,21^ These adaptations can align with infection control and use sanitized props. Staffing constraints may be addressed through brief, modular sessions delivered by trained personnel rather than dedicated specialists. Cost considerations, particularly in low-resource settings, support scalable strategies such as group sessions, integration into routine care, and low-cost digital or printed guides. These measures enhance feasibility while maintaining intervention fidelity.

Participants' interest in additional supportive services, like massage, counseling, and library access, highlights a notable gap in holistic cancer care, particularly in low- and middle-income countries.^22^ This finding aligns with evidence showing limited provision of psychosocial and supportive oncology services in such settings, although they can significantly enhance quality of life and symptom management.^22-26^ Variation in prior yoga exposure across sites reflects documented disparities in access to complementary and integrative health therapies, particularly among underserved populations. This supports the inclusion of structured orientations to increase understanding and engagement, as recommended in integrative oncology practice guidelines.^27^

Caregiver inclusion emerged as an important cultural component, consistent with findings that family engagement enhances emotional support and treatment adherence in cancer care, particularly in collectivist cultures.^28,29^ Patients at MNH and female patients at HCG preferred same-sex yoga therapists. This preference, heightened by the inpatient setting, reflects both modesty and cultural preference. In YT, attending to such sex-sensitive needs is important for ensuring patient comfort and engagement, and for using culturally appropriate adaptations of services in medical care.^30^ Also, introducing YT early during hospitalization and extending access postdischarge addresses key gaps in survivorship care,^31^ where yoga can be used as an evidence-based supportive intervention.

Finally, cultural adaptations, such as using neutral language to respect spiritual diversity and tailoring program introductions based on local norms, are essential yet under-addressed components of integrative therapies. Preserving core components (eg, breathwork, gentle movement, and meditation) while allowing contextual flexibility aligns with evidence-based principles for adapting behavioral interventions without compromising fidelity.

This study's multi-site, cross-cultural design provides valuable comparative insights into adapting yoga interventions across diverse health care settings, addressing a critical need for equity and contextual relevance in supportive cancer care. In-depth qualitative methods enriched understanding of patient and cultural nuances, supporting the expanding evidence base for adapted mind-body therapies. Limitations include a small sample size, limiting generalizability. However, thematic saturation occurred at each site by the fifth patient. There was also the potential for selection bias because of the purposeful recruitment from specialized centers. In addition, translation or interviewer-related variations may have existed, despite rigorous protocols

In conclusion, this study advances the understanding of how YT can be effectively adapted for HSCT patients across diverse cultural and clinical settings. The findings from this research directly informed modifications implemented in an ongoing single-arm, phase II clinical trial of yoga for patients undergoing HSCT at MDA (n = 15) and HCG (n = 15), ensuring that intervention adaptations were evidence-based and contextually appropriate.

As YT is recognized as an evidence-based component of cancer care, its thoughtful integration into all oncology settings and the larger global oncology practice can improve symptom management, emotional well-being, and overall quality of life. Our findings provide a replicable approach to cultural and contextual adaptation, one that promotes equity, engagement, and patient centeredness.
